# Supplementary material for: Plasma proteome profiling reveals dynamic of cholesterol marker after dual blocker therapy
Source: Nat Commun. 2024 May 8;15:3860. doi: 10.1038/s41467-024-47835-y (PMC11078984; doi:10.1038/s41467-024-47835-y)
Supplement: Supplementary file 1 — Supplementary Information [file 41467_2024_47835_MOESM1_ESM.pdf]

Plasma proteome profiling reveals dynamic of cholesterol marker after dual blocker therapy

Jiacheng Lyu[1#], Lin Bai[1#], Yumiao Li[2#], Xiaofang Wang[2#], Zeya Xu[1], Tao Ji[1], Hua Yang[2], Zizheng Song[2], Zhiyu Wang[2], Yanhong Shang[2], Lili Ren[2], Yan Li[3], Aimin Zang[2], Youchao Jia[2*], Chen Ding[1*]

Supplementary Figure 1

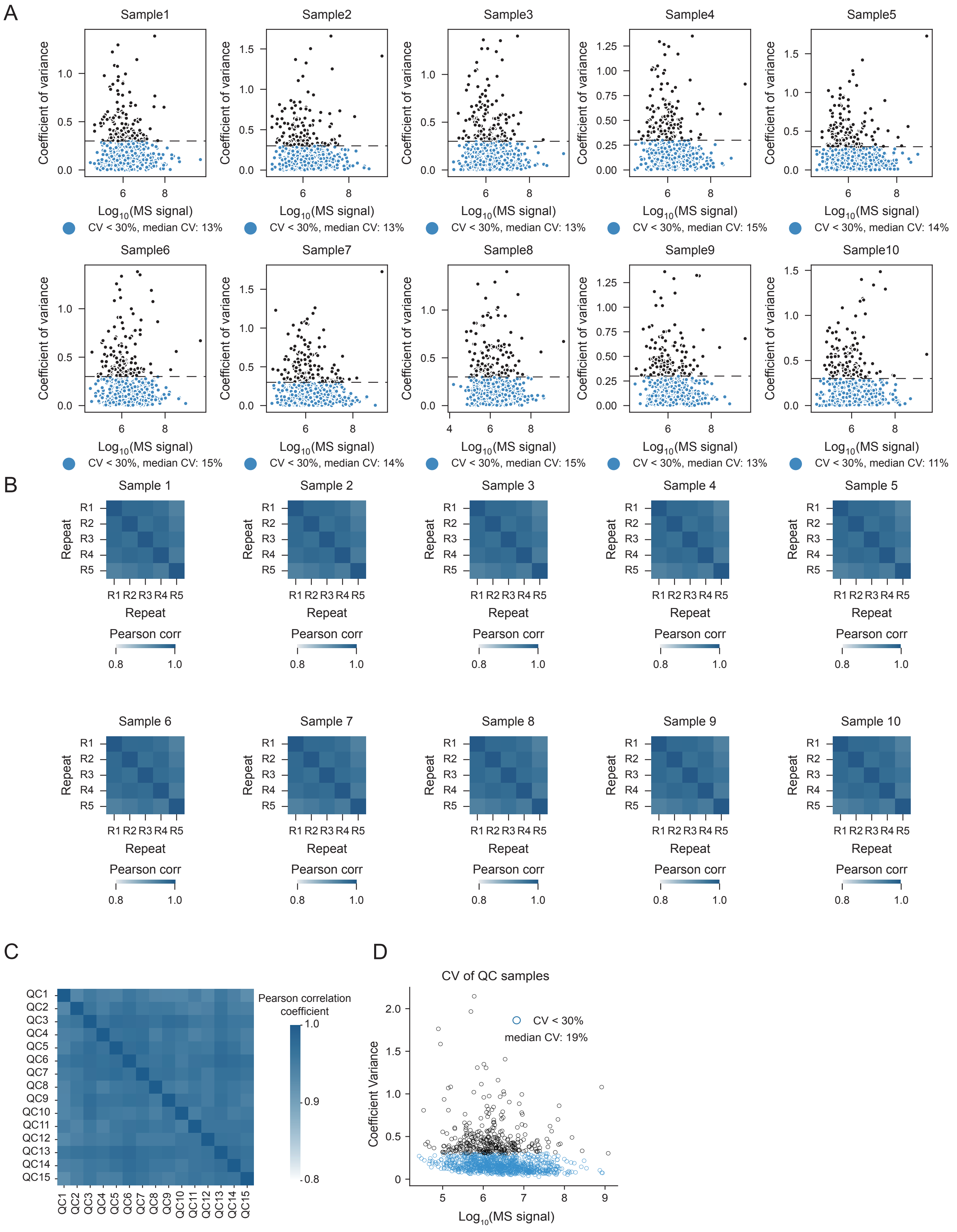

Supplementary Figure 1.

A. The scatter plot depicting the CVs among the 5 repeats for each of 10 samples. The blue dot indicated the CVs less than 30%.

B. The heatmap of the pearson correlation among 5 repeats for each of 10 samples.

C. Pearson correlation analysis of mixed QC samples.

D. Coefficient of variance of proteins of mixed QC samples.

Source data are provided as a Source Data file.

Supplementary Figure 2

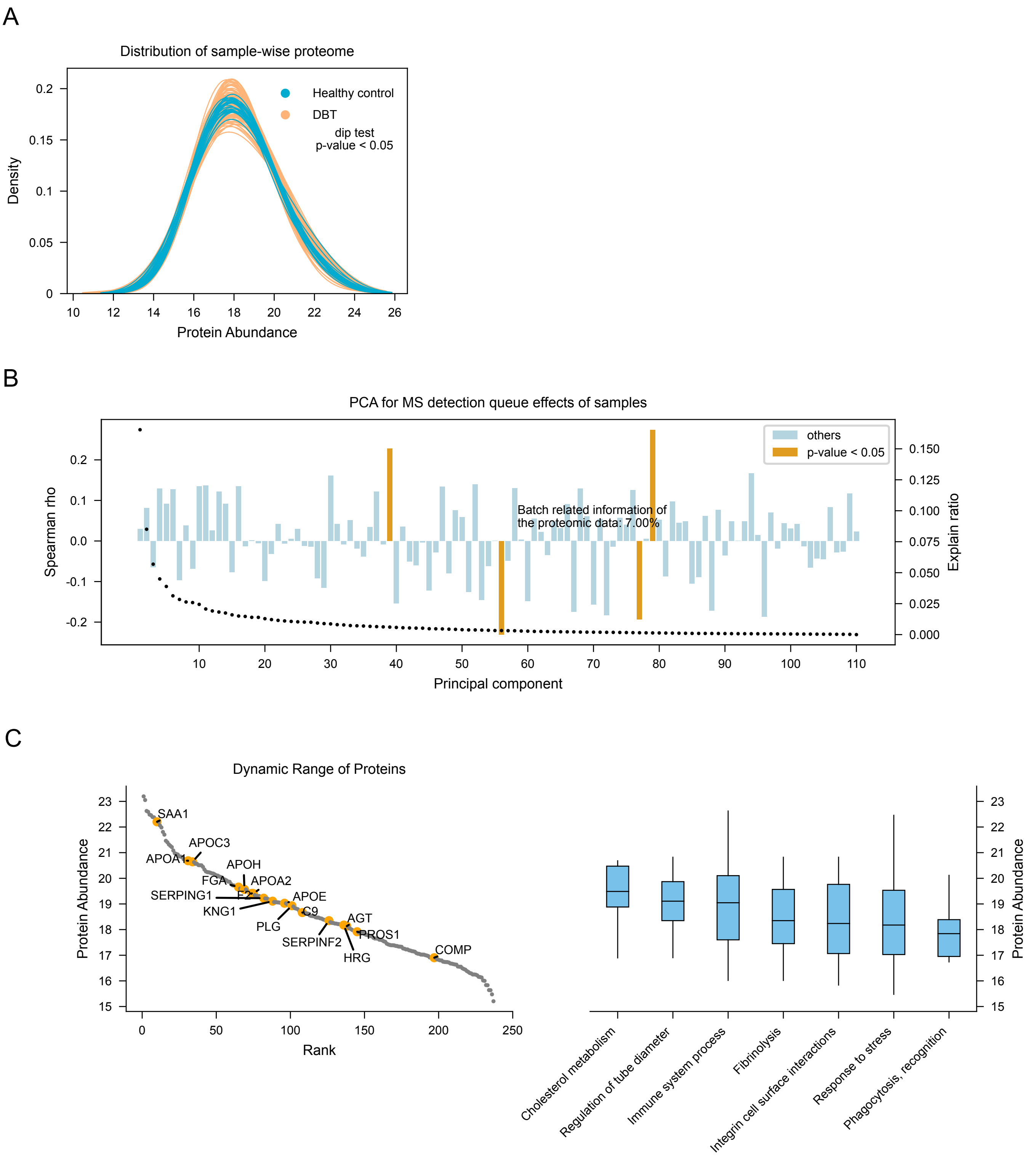

Supplementary Figure 2.

A. The density plot of healthy control (green line) and medicine therapy samples (orange line). The *p-values* were derived by two-sided dip test.

B. The batch effects evaluation of the plasma proteome dataset. The x-axis showed the principal components. The right y-axis (for the scatter plot) indicates the explained ratio of principal components. The left y-axis (for the bar plot) was the spearman correlation of batch effects variable and each principal component. The different color suggests the significance of the spearman correlation analysis.

C. Quantitative values of plasma proteins ranked according to their abundance. Several proteins are exemplified (orange dots). 7 enriched biological processes categories are highlighted as boxplots. The box ranges from the first (Q1) to the third quartile (Q3) of the distribution and represents the interquartile range (IQR). A line across the box indicates the median. The whiskers are lines extending from Q1 and Q3 to end points that defined as the most extreme data points within $Q1 - 1.5 \times IQR$ and $Q3 + 1.5 \times IQR$, respectively.

Source data are provided as a Source Data file.

Supplementary Figure 3

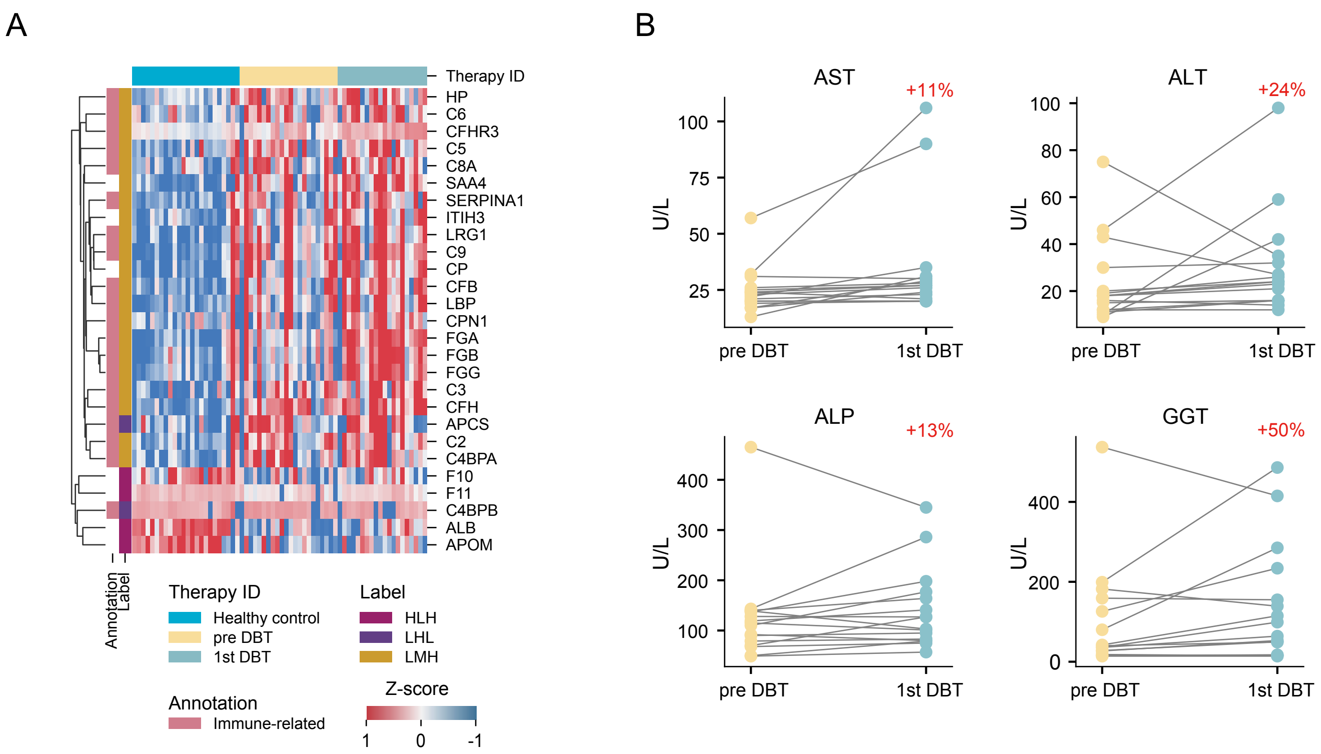

Supplementary Figure 3.

A. The heatmap of 27 differently expressed proteins (HLH, LHL, LMH) which annotated as liver-specific proteins among health control group, pre DBT group, and 1st DBT cycle group. Values were transformed by z-score.

B. Pair-wised level of four liver function damage related blood routines between pre DBT (n = 16) and first DBT samples (n = 16). AST, Aspartate transaminase; ALT, Alanine aminotransferase; ALP, alkaline phosphatase; GGT, Gamma-glutamyl transferase.

Source data are provided as a Source Data file.

Supplementary Figure 4

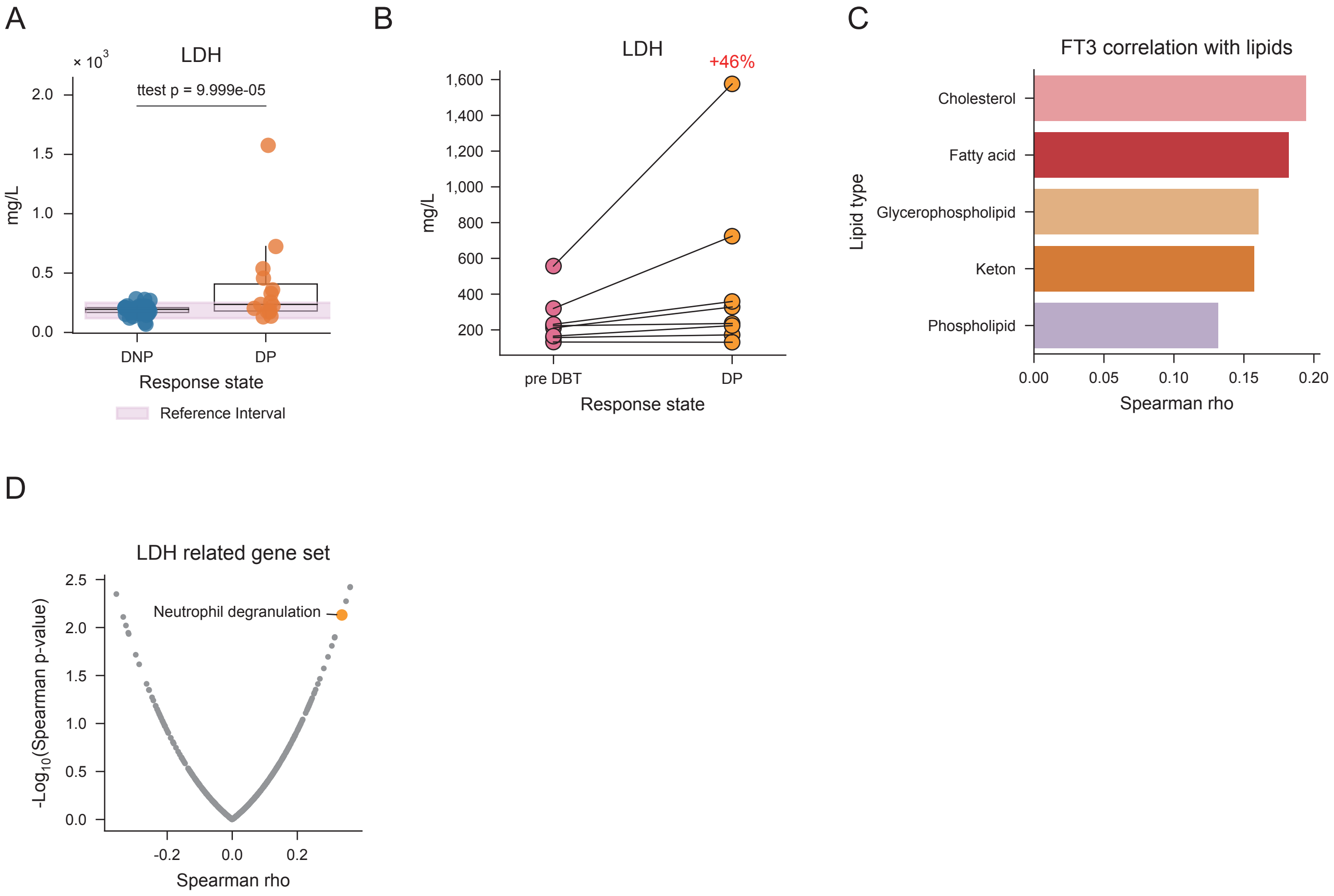

Supplementary Figure 4.

A. The boxplot describes the level of lactate dehydrogenase (LDH) between disease non-progressive (DNP) (n = 47) and disease progressive (DP) (n = 15) samples at blood routine level. The pink rectangle shows the normal reference interval. *P-value* was derived by two-sided permutation-based t-test. The box ranges from the first (Q1) to the third quartile (Q3) of the distribution and represents the interquartile range (IQR). A line across the box indicates the median. The whiskers are lines extending from Q1 and Q3 to end points that defined as the most extreme data points within Q1 − 1.5 × IQR and Q3 + 1.5 × IQR, respectively.

B. Pair-wised plot of LDH at blood routine level between pre DBT samples (n = 8) and DP samples (n = 8). *P-value* was derived by two-sided paired t-test.

C. Bar plot depicting the spearman correlation of FT3 and different type of lipids including cholesterol, fatty acid, glycerophospholipid, ketone, and phospholipid.

D. Scatterplot depicting the correlation of LDH and gene set ssGSEA scores. Neutrophil degranulation was exemplified by orange dots.

Source data are provided as a Source Data file.

Supplementary Figure 5

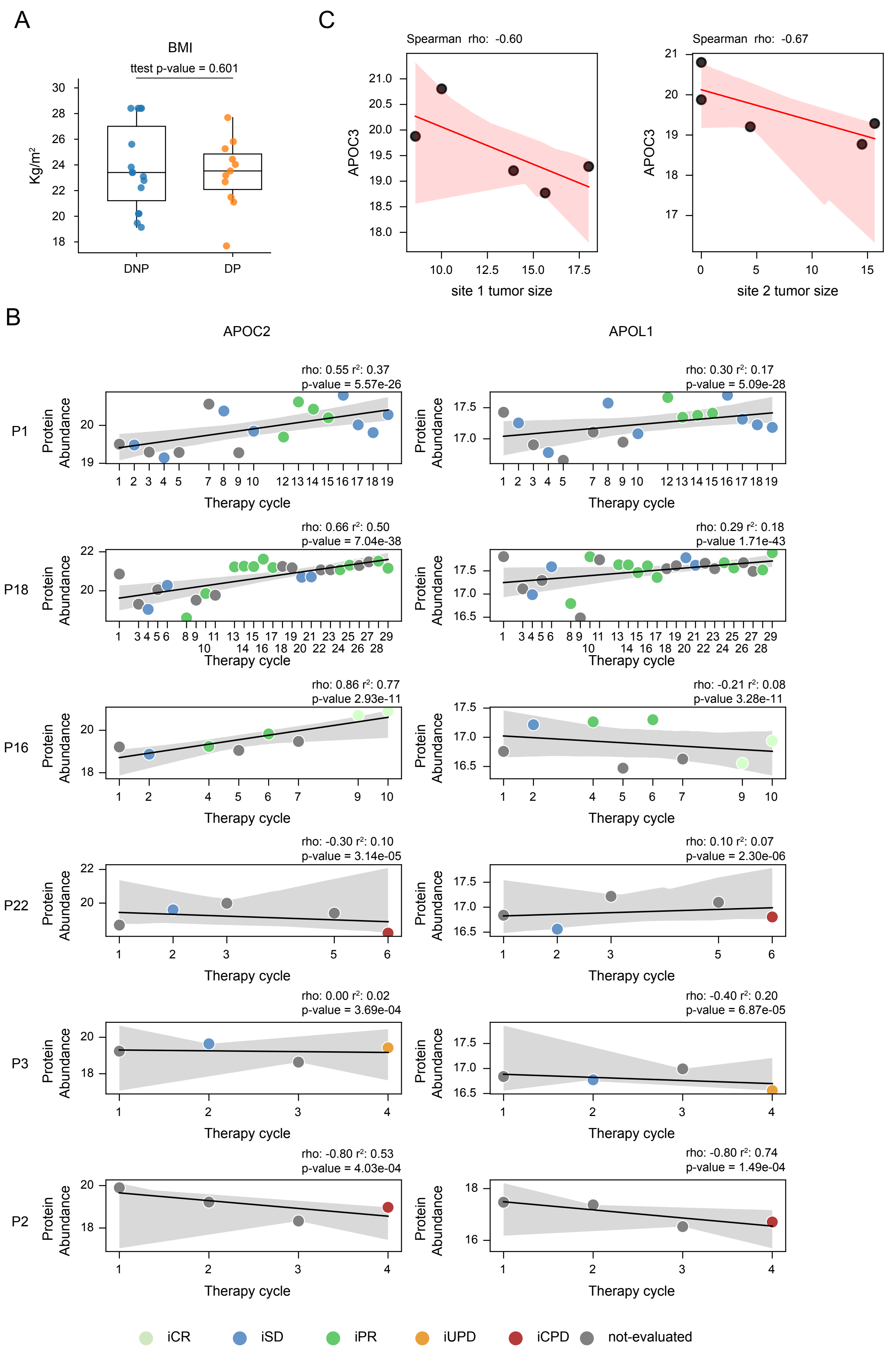

Supplementary Figure 5.

A. The box plot depicting the body mass index (BMI) between DNP samples (n = 30) and DP samples (n = 15). *P-values* were derived by two-sided permutation-based t-test. The box ranges from the first (Q1) to the third quartile (Q3) of the distribution and represents the interquartile range (IQR). A line across the box indicates the median. The whiskers are lines extending from Q1 and Q3 to end points that defined as the most extreme data points within Q1 − 1.5 × IQR and Q3 + 1.5 × IQR, respectively.

B. The panel shows the time series-linear regression of APOC2 and APOL1 in 6 patients (P1, P16, P18 with all DNP samples, and P2, P3, P22 with DP samples), separately. Dot color represents the evaluated response state. The translucent bands around the regression line indicates the 95% confidence interval. *P-values* were derived by the two-sided F test.

C. The negative correlation of APOC3 protein level and two metastases tumor size in P16, separately. The translucent bands around the regression line indicates the 95% confidence interval.

Source data are provided as a Source Data file.

Supplementary Figure 6

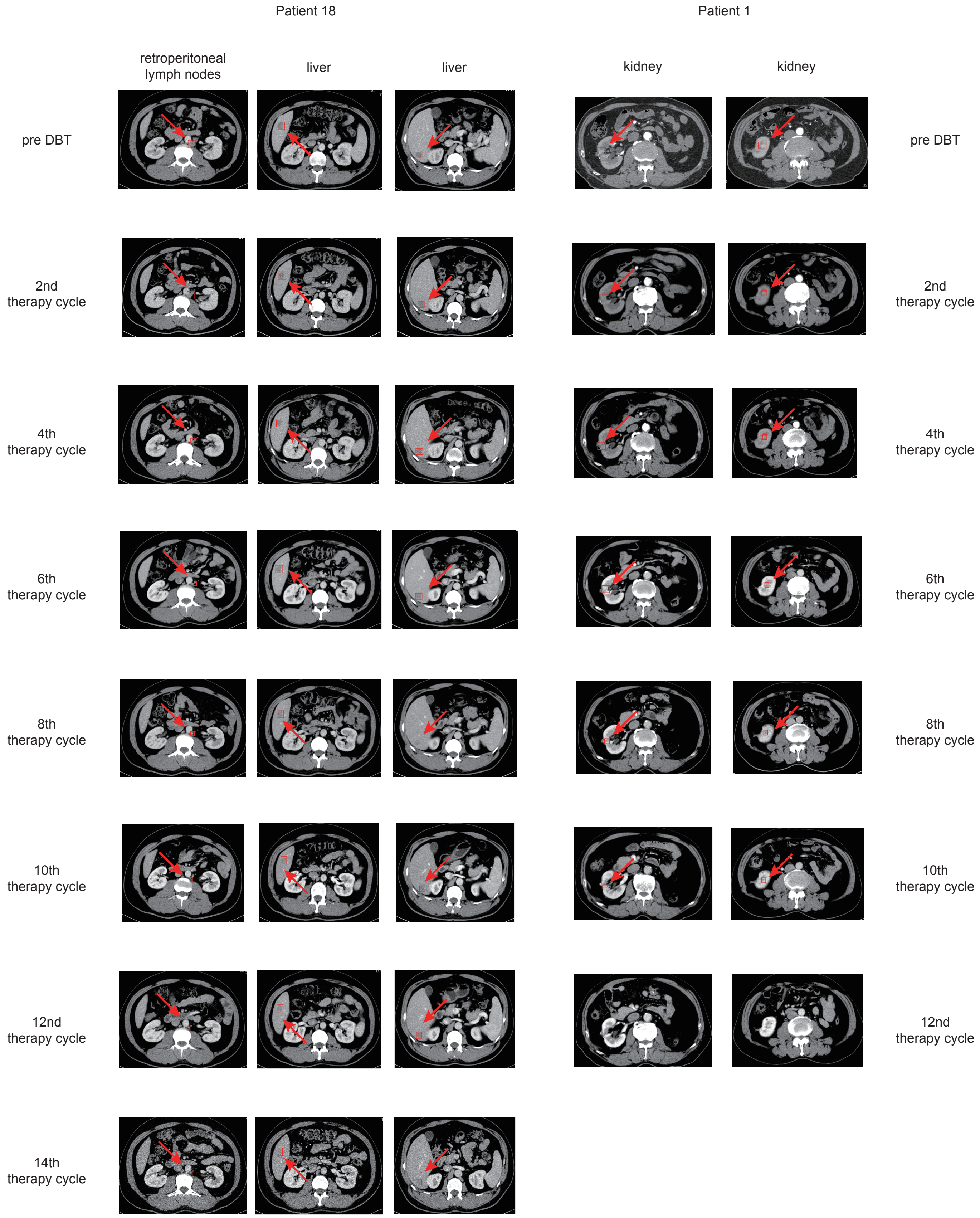

Supplementary Figure 6.

The tumor sites radiology imaging of P1 and P18 with longitudinal samples, separately.

Supplementary Figure 7

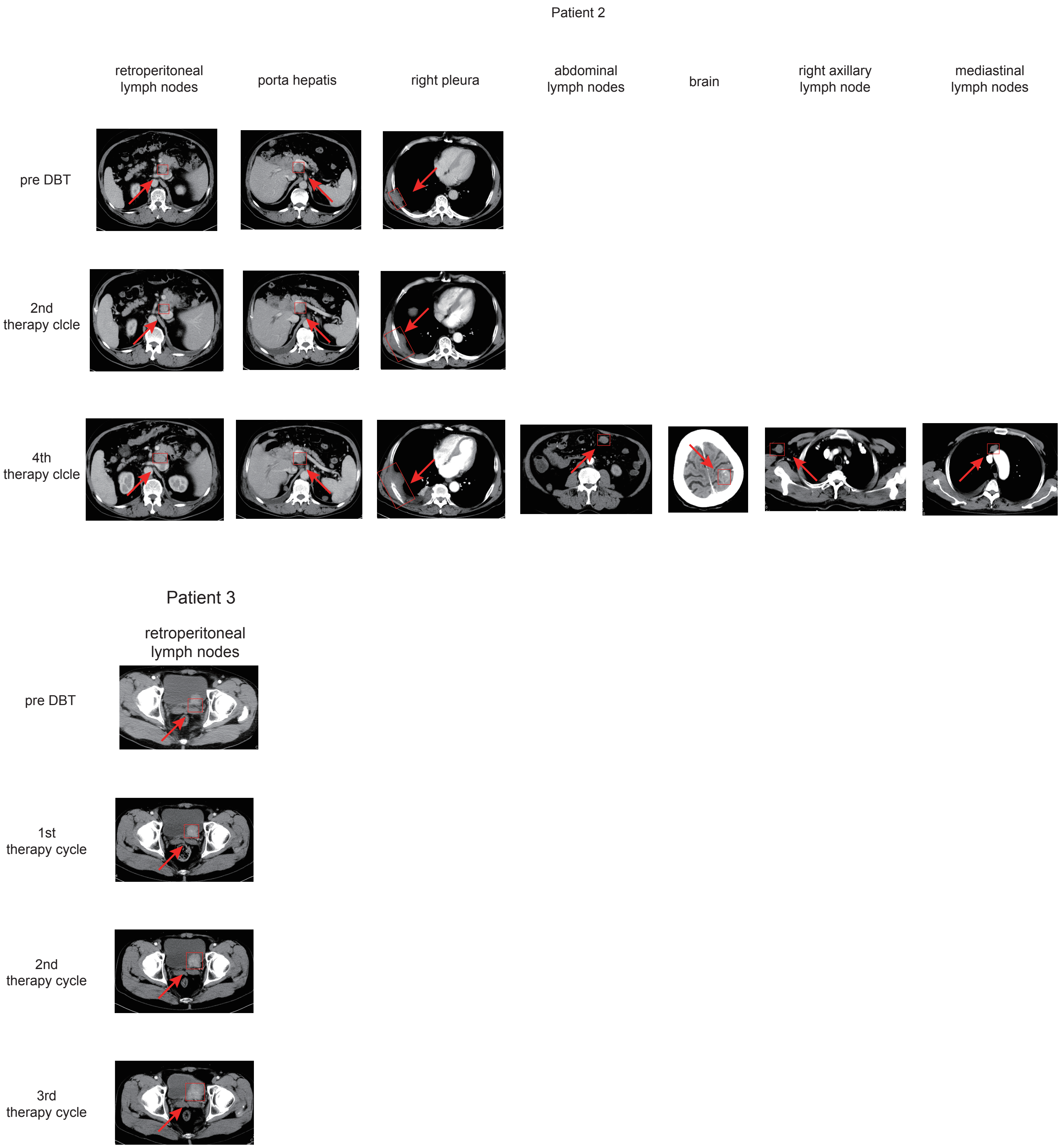

Supplementary Figure 7.

The tumor sites radiology imaging of P2 and P3 with longitudinal samples, separately.

Supplementary Figure 8

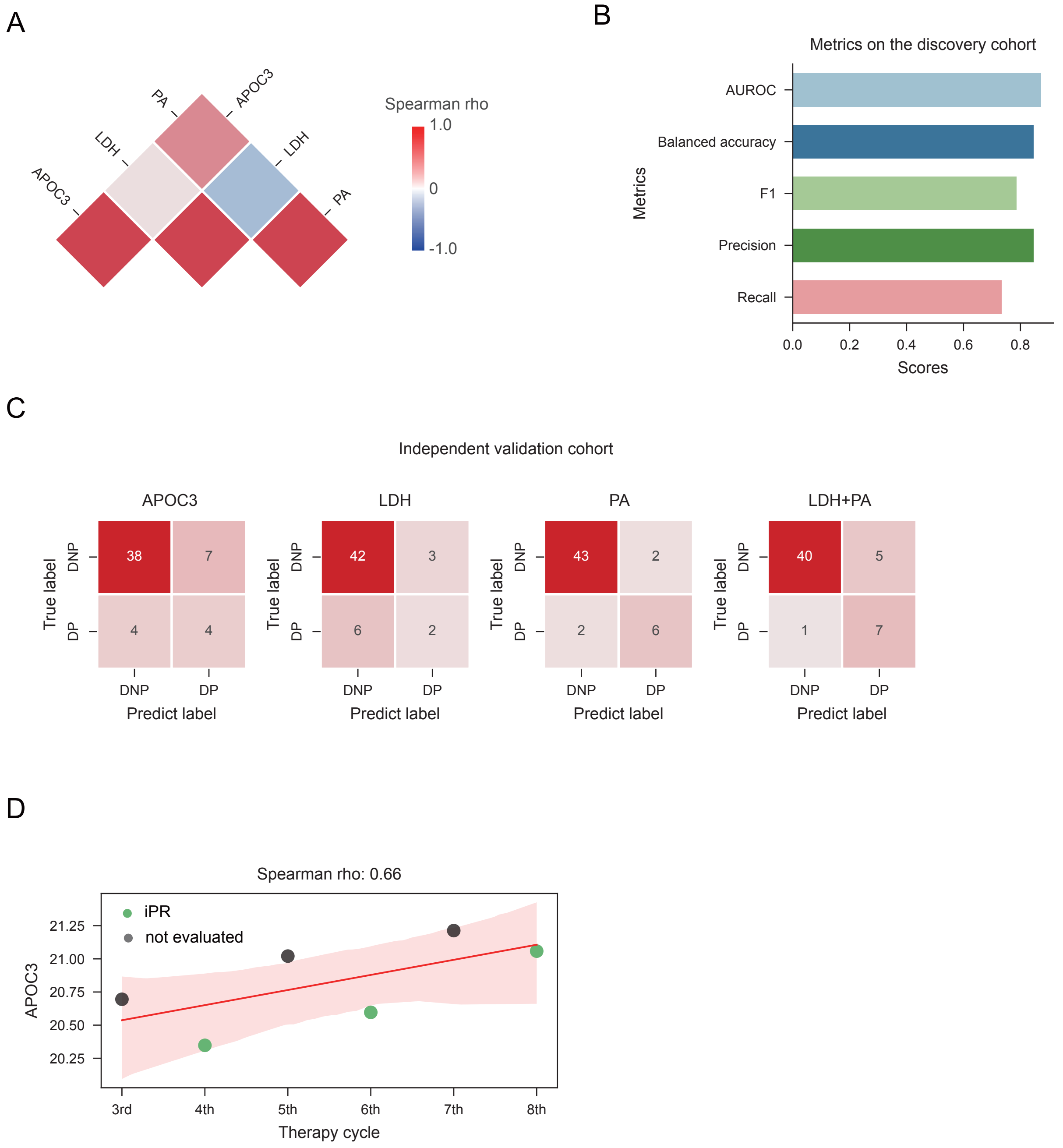

Supplementary Figure 8.

A. The heatmap depicting the collinearity of three features LDH, PA, and APOC3 used in the machine learning model on the discovery cohort.

B. The bar plot depicting the model performance on the discovery cohort by evaluating the five metrics including area under the ROC curve (AUROC), balanced accuracy, F1, precision, and recall.

C. The confusion matrix of the models with different feature combinations on the independent validation cohort.

D. The APOC3 protein level in a responded patient with 6 samples in the independent validation cohort. The translucent bands around the regression line indicates the 95% confidence interval.

Source data are provided as a Source Data file.
