# Supplementary material for: Plasma proteome profiling reveals dynamic of cholesterol marker after dual blocker therapy
Source: Nat Commun. 2024 May 8;15:3860. doi: 10.1038/s41467-024-47835-y (PMC11078984; doi:10.1038/s41467-024-47835-y)
Supplement: Supplementary file 3 — Reporting Summary [file 41467_2024_47835_MOESM3_ESM.pdf]

nature portfolio

Corresponding author(s): Chen Ding, Youchao Jia

Last updated by author(s): Mar 16, 2024

Reporting Summary

Nature Portfolio wishes to improve the reproducibility of the work that we publish. This form provides structure for consistency and transparency in reporting. For further information on Nature Portfolio policies, see our Editorial Policies and the Editorial Policy Checklist.

Statistics

For all statistical analyses, confirm that the following items are present in the figure legend, table legend, main text, or Methods section.

n/a	Confirmed	
☐	☒	The exact sample size (*n*) for each experimental group/condition, given as a discrete number and unit of measurement
☐	☒	A statement on whether measurements were taken from distinct samples or whether the same sample was measured repeatedly
☐	☒	The statistical test(s) used AND whether they are one- or two-sided<br>*Only common tests should be described solely by name; describe more complex techniques in the Methods section.*
☐	☒	A description of all covariates tested
☐	☒	A description of any assumptions or corrections, such as tests of normality and adjustment for multiple comparisons
☐	☒	A full description of the statistical parameters including central tendency (e.g. means) or other basic estimates (e.g. regression coefficient) AND variation (e.g. standard deviation) or associated estimates of uncertainty (e.g. confidence intervals)
☐	☒	For null hypothesis testing, the test statistic (e.g. *F*, *t*, *r*) with confidence intervals, effect sizes, degrees of freedom and *P* value noted<br>*Give P values as exact values whenever suitable.*
☒	☐	For Bayesian analysis, information on the choice of priors and Markov chain Monte Carlo settings
☒	☐	For hierarchical and complex designs, identification of the appropriate level for tests and full reporting of outcomes
☐	☒	Estimates of effect sizes (e.g. Cohen's *d*, Pearson's *r*), indicating how they were calculated

Our web collection on statistics for biologists contains articles on many of the points above.

Software and code

Policy information about availability of computer code

Data collection	Plasma samples were analyzed on a Q Exactive HF-X mass spectrometer (Thermo Fisher Scientific, Rockford, IL, USA) coupled with high-performance liquid chromatography (EASY-nLC 1200 System, Thermo Fisher Scientific).The mass spectrometry data were acquired using the Xcalibur software v2.2 (Thermo Fischer Scientific).
Data analysis	The mass spectrometry raw files were processed in Firmiana, searched against the UniProt human protein database (reviewed sequences only; as updated on 2019.12.17, housing 20,406 entries). Statistical analyses were realized by python (v3.9) or R (v4.0). Approaches or algorithms used for the proteome data annotation include pandas (v1.5.3), numpy (v1.26.3), scipy (v1.12.0), statsmodels (v0.14.1), scikit-learn (v1.2.1). Gene annotation was performed using gprofiler (v1.0.0). Standard statistical tests were used to analyze the clinical and proteome data, including but not limited to permutation-based student's t test, Kruskal-Wallis test, Spearman's correlation and Pearson's correlation test. Unless otherwise specified, all statistical tests were two-sided, and statistical significance was considered when p value < 0.05. To account for multiple-testing, the p values were adjusted using the Benjamini–Hochberg FDR correction. All the analyses of clinical and proteome data were performed in python and R. The code used for this study was deposited at GitHub (https://github.com/Jiacheng-Lyu/DBT-plasma-proteome).The code used for this study was deposited at GitHub (https://github.com/Jiacheng-Lyu/DBT-plasma-proteome). The code was also deposited at Zenodo (https://zenodo.org/doi/10.5281/zenodo.10824474).

For manuscripts utilizing custom algorithms or software that are central to the research but not yet described in published literature, software must be made available to editors and reviewers. We strongly encourage code deposition in a community repository (e.g. GitHub). See the Nature Portfolio guidelines for submitting code & software for further information.

Data

Policy information about availability of data

All manuscripts must include a data availability statement. This statement should provide the following information, where applicable:

- Accession codes, unique identifiers, or web links for publicly available datasets
- A description of any restrictions on data availability
- For clinical datasets or third party data, please ensure that the statement adheres to our policy

The proteome raw datasets have been deposited to the ProteomeXchange Consortium (dataset identifier: PXD039260) via the iProX partner repository (https://www.iprox.cn/) under Project ID: IPX0005695001 (https://www.iprox.cn/page/PDV0141.html).
In detail, the data of the discovery cohort and independent validation cohort were uploaded, separately:
1) Annotation file: the annotation of sample information including sample name, therapy cycle, etc.;
2) MS raw data: the MS raw data;
3) FASTA file: the FASTA file used for the MS data processing;
4) DIA-NN output file: the merged DIA-NN standard output file;
5) Proteome expression matrix: the quantification matrix data.
The more detailed description could be found in the README file under the above link.
The remaining data are available within the Article, Supplementary Information. Source data are provided with this paper.

Research involving human participants, their data, or biological material

Policy information about studies with human participants or human data. See also policy information about sex, gender (identity/presentation), and sexual orientation and race, ethnicity and racism.

Reporting on sex and gender	There were 49 patients in the cohort, 33 males and 16 females.
Reporting on race, ethnicity, or other socially relevant groupings	This study included 49 Chinese patients with tumors at the Affiliated Hospital of Hebei University, of which 22 patients were treated with PD1/CTLA4 dual blocker and 27 patients were treated with PD1 blocker.
Population characteristics	This study included 49 Chinese patients with tumors at the Affiliated Hospital of Hebei University. For the discovery cohort, 22 patients with tumors were enrolled, who were treated with PD1/CTLA4 dual blocker or PD1 blocker, with an age range 35-71.
Recruitment	Participants with blood samples at enrollment were identified for this study from previous PD1/CTLA4 dual blocker cohorts. The plasma samples were collected before each therapy cycle, and the efficacy of the therapy were evaluated per two cycles. In total, 113 samples of 22 patients with tumor were enrolled for the analysis.
Ethics oversight	The study was compliant with the ethical standards of Helsinki Declaration II and was approved by the institutional review board of Affiliated Hospital of Hebei University (HDFY-LL-2020-157). Written informed consent was obtained from each patient before any study-specific investigation was conducted.

Note that full information on the approval of the study protocol must also be provided in the manuscript.

Field-specific reporting

Please select the one below that is the best fit for your research. If you are not sure, read the appropriate sections before making your selection.

☒ Life sciences ☐ Behavioural & social sciences ☐ Ecological, evolutionary & environmental sciences

For a reference copy of the document with all sections, see nature.com/documents/nr-reporting-summary-flat.pdf

Life sciences study design

All studies must disclose on these points even when the disclosure is negative.

Sample size	We collected the plasma samples based on strict criteria from 22 Chinese patients treated with PD1/CTLA4 dual blocker (as the discovery cohort) and 27 Chinese patients treated with PD1 blocker (as the independent validation cohort). For the discovery cohort, we collected the longitudinal samples before each therapy cycle. Total of 113 samples were collected for the discovery cohort. No statistical method was used to predetermine sample size. Referring to the breast cancer study (PMID: 32265444) and the plasma proteome based non-alcoholic fatty liver disease study (PMID: 30824564), we thought that sample size of 113 is sufficient for this study.
Data exclusions	One sample in the independent validation cohort was excluded for the downstream analysis due to not pass the quality control during the proteome quantification.
Replication	Strict significance thresholds were applied in this analysis. The classifier performance were validated in the independent validation cohort. In order to evaluate the reproducibility of the MS detection for the actual samples, we had randomly selected 10 actual samples and detected in

	consecutive replicates 5 times for each sample. The high correlation and low coefficient of variance among the repeated actual samples indicated the stability and robustness of the MS platform.
Randomization	For ESI-LC-MS/MS analysis, the longitudinal samples from different patient and different therapy cycle were detected with a random order to exclude the bias effects of the mass spectrometry. As for the machine learning model construction, the samples were randomly divided into train and test cohort.
Blinding	The investigators who measured protein expression were blinded to patient information. For sample processing, PCA, linear regression, AB group testing, all investigators were blinded to clinical information (including TNM stage, and patients outcomes).

Reporting for specific materials, systems and methods

We require information from authors about some types of materials, experimental systems and methods used in many studies. Here, indicate whether each material, system or method listed is relevant to your study. If you are not sure if a list item applies to your research, read the appropriate section before selecting a response.

Materials & experimental systems

n/a	Involved in the study
☒	☐ Antibodies
☒	☐ Eukaryotic cell lines
☒	☐ Palaeontology and archaeology
☒	☐ Animals and other organisms
☒	☐ Clinical data
☒	☐ Dual use research of concern
☒	☐ Plants

Methods

n/a	Involved in the study
☒	☐ ChIP-seq
☒	☐ Flow cytometry
☒	☐ MRI-based neuroimaging

Plants

Seed stocks	*Report on the source of all seed stocks or other plant material used. If applicable, state the seed stock centre and catalogue number. If plant specimens were collected from the field, describe the collection location, date and sampling procedures.*
Novel plant genotypes	*Describe the methods by which all novel plant genotypes were produced. This includes those generated by transgenic approaches, gene editing, chemical/radiation-based mutagenesis and hybridization. For transgenic lines, describe the transformation method, the number of independent lines analyzed and the generation upon which experiments were performed. For gene-edited lines, describe the editor used, the endogenous sequence targeted for editing, the targeting guide RNA sequence (if applicable) and how the editor was applied.*
Authentication	*Describe any authentication procedures for each seed stock used or novel genotype generated. Describe any experiments used to assess the effect of a mutation and, where applicable, how potential secondary effects (e.g. second site T-DNA insertions, mosiacism, off-target gene editing) were examined.*
