# Supplementary material for: Plasma proteome profiling reveals dynamic of cholesterol marker after dual blocker therapy
Source: Nat Commun. 2024 May 8;15:3860. doi: 10.1038/s41467-024-47835-y (PMC11078984; doi:10.1038/s41467-024-47835-y)
Supplement: Supplementary file 4 — Description of Additional Supplementary Files [file 41467_2024_47835_MOESM4_ESM.pdf]

Description of Additional Supplementary Files

Supplementary Data 1

Description: **Clinical and proteome characteristics of the DBT cohort. (a)** Baseline Demographic and Disease Characteristics of Patients in Dual Blocker Therapy (DBT) cohort. **(b)** The protein concentration of the samples in DBT cohort. **(c)** Clinical characteristics of samples in DBT cohort. **(d)** Protein expression matrix of DBT cohort.

Supplementary Data 2

Description: **The effects of DBT on the plasma proteome. (a)** The z-score value of the proteome data. **(b)** The statistic table of proteome data among healthy control group, before DBT group and its matched 1st DBT group. **(c)** The gene set enrichment results for the different protein clusters.

Supplementary Data 3

Description: **The differential analysis of clinical indicators for the DBT cohort. (a)** The statistic table of blood routines between non-disease progression group and disease progression group. **(b)** The correlation table of blood routine FT3 and ssGSEA gene set scores. **(d)** The correlation table of blood routine LDH and ssGSEA gene set scores. **(d)** The correlation table of blood routine PA and ssGSEA gene set scores.

Supplementary Data 4

Description: **The differential analysis of proteome for the DBT cohort. (a)** The statistic table of ssGSEA scores between non-disease progression group and disease progression group. **(b)** The statistic table of protein expression between non-disease progression group and disease progression group.

Supplementary Data 5

Description: **The machine learning model for the DBT cohort. (a)** Feature importance of machine learning model with integrated all features. **(b)** Features of independent validation anti-PD1 monotherapy cohort.
